# ABO blood type is associated with ovarian reserve in Chinese women with subfertility

**DOI:** 10.18632/oncotarget.10766

**Published:** 2016-07-22

**Authors:** Liangshan Mu, Wumin Jin, Haiyan Yang, Xia Chen, Jiexue Pan, Jia Lin, Peiyu Wang, Xuefeng Huang

**Affiliations:** ^1^ Reproductive Medicine Center, The First Affiliated Hospital of Wenzhou Medical University, Wenzhou, People's Republic of China

**Keywords:** blood type, FSH, infertility, ovarian reserve, Pathology Section

## Abstract

Ovarian reserve reflects both the quantity and quality of oocytes available for procreation, and is affected by many known and unknown factors. ABO blood type is related to a number of infertility processes, but it is unclear whether and how ABO blood type affects ovarian reserve. Here, we explored the relationship between ABO blood type and ovarian reserve in Chinese women with subfertility. Day-3 serum follicle-stimulating hormone (FSH) levels and blood type were examined in 14,875 women who underwent IVF or ICSI treatment. Blood type proportions in the patient population were as follows: 30.98% type A, 24.54% type B, 7.57% type AB, and 36.91% type O. A higher percentage of women with diminished ovarian reserve (DOR) were blood type O, while a lower percentage had the B antigen (B and AB). Multiple logistic regression analysis revealed that blood type O was associated with a greater risk of DOR than blood type B and B antigen-positive types. By contrast, the B antigen (B and AB) was associated with a lower incidence of DOR than blood type O. These results suggest that blood type O is a risk factor for DOR while the B antigen (blood type B or AB) is a protective factor for ovarian reserve in Chinese women with subfertility. Further studies are needed to confirm this effect and identify the underlying mechanisms.

## INTRODUCTION

Ovarian reserve, a measure that takes into account the quantity and quality of remaining oocytes, is indicative of a woman's reproductive potential [1]. Assessment of ovarian reserve, which involves testing antral follicle count (AFC) and follicle stimulating hormone (FSH), anti-mullerian hormone (AMH), and inhibin-B levels [2], is helpful for women who want to achieve pregnancy. Generally, an early follicular phase serum FSH concentration > 10 IU/L indicates an increased risk of diminished ovarian reserve (DOR) [3–5]. Many factors are related to DOR, including age, ovarian surgery, endometriosis, chemotherapy, and abdominal radiation [6, 7].

Recent studies have examined the relationship between blood type and ovarian reserve, but have obtained contradictory results [8–12]. Among these studies, two indicated that blood type is associated with ovarian reserve, although the directions of the relationships were inconsistent [8, 9]. The other three studies found no relationship between blood type and ovarian reserve [10–12]. Additional studies are necessary to reconcile these conflicting findings. We therefore collected and analyzed data from our center to investigate the association between blood type and ovarian reserve.

## RESULTS

### Baseline characteristics of the study population

The mean subject age was 31.11 ± 4.37 years, and the mean FSH level was 8.28 ± 3.10 IU/L. The mean E2 level, which was measured at the same time as FSH, was 166.1 ± 133.4 pmol/L. 30.98% of patients were blood type A, 24.54% were type B, 7.57% were type AB, and 36.91% were type O. Other general demographic and clinical characteristics of the study population are summarized in Table 1.

**Table 1:** Charactersitics of study population

Variables	
Number of cases	14875
Age, years	31.1 ± 4.4
BMI, Kg/m2	21.4 ± 2.8
FSH, IU/L	8.3 ± 3.1
E2, pmol/L	166.1 ± 133.4
AFC, n	15.6 ± 7.5
Blood type	
A, n (%)	4608 (31.0)
B, n (%)	3651 (24.5)
AB, n (%)	1126 (7.6)
O, n (%)	5490 (36.9)
Subfertily	
Duration, years	4 (2-6)
Primary subfertilty, n (%)	4527 (30.4)
Secondary subfertilty, n (%)	10348 (69.6)
History	
Endometriosis, n (%)	794 (5.3)
Ovarian surgery, n (%)	964 (6.5)

Data are shown as mean ± SD or number (percentage).

Abbreviations: BMI presents body mass index; FSH, follicle stimulating hormone; E2, estrogen2; AFC, antral follicle count.

### Clinical characteristics and blood type proportions according to ovarian reserve status

As shown in Table 2, participants were divided into two groups based on FSH concentration. Subjects with ≤ 10 IU/L FSH were assigned to group Ι (*n* = 12286), and those with > 10 IU/L FSH were assigned to group II (*n* = 2589). Women in group Ι were younger (30.82 ± 4.22 years *vs*. 32.52 ± 4.80 years; *p* < 0.001) and had higher BMIs (21.47 ± 2.87 kg/m^2^ *vs*. 20.87 ± 2.61 kg/m^2^; *p* < 0.001) than those in group II. Compared to group II, women in group Ι had higher AFCs (16.51 ± 7.50 *vs*. 11.05 ± 5.66; *p* < 0.001) and shorter durations of subfertility (4.39 ± 3.22 *vs*. 4.80 ± 3.74; *p* < 0.001). There were no significant differences in histories of endometriosis and ovarian surgery between the groups. The proportions of blood type B (24.91% *vs*. 22.79%; *p* = 0.02) and B antigen (B and AB) (32.66% *vs*. 29.55%; *p* = 0.002) were higher in group Ι than in group II. In contrast, a lower percentage of group Ι women were blood type O (36.46% *vs*. 39.01%; *p* = 0.02) compared to group II. There were no significant differences in the proportions of other blood types between the groups.

**Table 2:** Characteristics of study population according to ovarian reserve status

Variables	Group I	Group II	
	FSH ≤10 IU/L	FSH >10IU/L	*P*
N	12286	2589	
Age, years	30.82 ± 4.22	32.52 ± 4.80	<0.001
BMI, Kg/m^2^	21.47 ± 2.87	20.87 ± 2.61	<0.001
AFC, n	16.51 ± 7.50	11.05 ± 5.66	<0.001
E2, pmol/L	167.35 ± 137.24	160.08 ± 113.36	0.02
Duration of subfertility, years	4.39 ± 3.22	4.80 ± 3.74	<0.001
Endometriosis, n (%)	662 (5.39)	132 (5.10)	0.55
Ovarian surgery, n (%)	794 (6.46)	170 (6.57)	0.85
Blood type			
A, n (%)	3794 (30.88)	814 (31.44)	0.58
B, n (%)	3061 (24.91)	590 (22.79)	0.02
AB, n (%)	951 (7.74)	175 (6.76)	0.09
O, n (%)	4480 (36.46)	1010 (39.01)	0.02
A antigen (A and AB), n (%)	4745 (38.62)	989 (38.20)	0.69
B antigen (B and AB), n (%)	4012 (32.66)	765 (29.55)	0.002

Data are shown as mean ± SD or number (percentage).

Abbreviations: BMI presents body mass index; FSH, follicle stimulating hormone; E2, estrogen2; AFC, antral follicle count.

### Association between blood type and diminished ovarian reserve

As shown in Table 3, after adjusting for age, BMI, AFC, subfertility types, duration of subfertility, history of endometriosis and ovarian surgery, and blood type A, multivariate logistic regression revealed that blood type O was associated with increased risk of DOR (OR = 1.45, 95% = 1.21-1.75, *p* < 0.001, relative to blood type B; OR = 1.46, 95% = 1.23-1.74, *p* < 0.001, relative to blood types B and AB). In contrast, both blood type B and the B antigen (B and AB) were associated with an obvious decrease in the incidence of DOR (OR = 0.69, 95% = 0.57-0.83, *p* < 0.001, and OR = 0.68, 95% = 0.57-0.82, *p* < 0.001, respectively, both relative to blood type O). In addition, age and subfertility type were associated with an elevated occurrence of DOR (OR = 1.03, 95% = 1.02-1.05, *p* < 0.001, and OR = 1.33, 95% = 1.12-1.59, *p =* 0.001, respectively). Conversely, BMI and AFC were negatively associated with DOR (OR = 0.91, 95% = 0.90-0.93, *p* < 0.001, and OR = 0.87, 95% = 0.86-0.88, *p* < 0.001, respectively). There were no significant associations between duration of subfertility or histories of endometriosis and ovarian surgery and the incidence of DOR.

**Table 3:** Multivariate logistic regresssion between clinical characteristics and blood type on diminished ovarian reserve

Variables	OR (95% CI)	*P*
Age	1.03 (1.02-1.05)	<0.001
BMI	0.91 (0.90-0.93)	<0.001
AFC	0.87 (0.86-0.88)	<0.001
Subfertility types	1.33 (1.12-1.59)	0.001
Duration of subfertility	1.00 (0.98-1.01)	0.48
Endometriosis	0.91 (0.74-1.11)	0.35
Ovarian surgery	1.05 (0.87-1.26)	0.62
Blood type		
O^a^	1.45 (1.21-1.75)	<0.001
O^b^	1.46 (1.23-1.74)	<0.001
B^c^	0.69 (0.57-0.83)	<0.001
B antigen (B and AB)^d^	0.68 (0.57-0.82)	<0.001

a blood type B as the reference group

b B antigen (B and AB) as the reference group

c, d blood type O as the reference group.

Abbreviations: BMI presents body mass index; FSH, follicle stimulating hormone; AFC, antral follicle count; OR, odds ratio; CI, confidence interval.

## DISCUSSION

We performed a retrospective study in Chinese women with subfertility undergoing cycles of IVF or ICSI. Our analysis indicated that ABO blood type was closely associated with ovarian reserve in these patients; those with blood type O had an increased risk of DOR, while the B antigen (blood type B or AB) was associated with a decreased incidence of DOR.

DOR, defined as a decreased quantity and quality of oocytes, affects nearly 10% of women seeking fertility treatment [13]. Multiple pathophysiologic factors affect ovarian reserve, including age, autoimmune conditions, ovarian surgery, chemotherapy, radiation, and genetics [6, 7]. Several recent studies explored the association between ABO blood type and ovarian reserve. However, the results were inconsistent; as shown in Table 4, two studies found an association between blood type and ovarian reserve. Both studies examined FSH levels to evaluate ovarian reserve and used the same definition for DOR. Nejat *et al*. [8] observed that women with blood type O were twice as likely to have increased baseline FSH concentrations compared to women with blood types A or AB. Additionally, blood type O was associated with an increased risk of DOR, while the A antigen (blood type A or AB) was associated with a reduced risk. In contrast, Lin *et al*. [9] found that Chinese women with blood type O were less likely to have DOR, while the B antigen (blood type B or AB) was a risk factor for DOR. Blood type A was not related to ovarian reserve in that study. Finally, the remaining three studies found no association between blood type and ovarian reserve. These conflicting findings may be due to racial variation between the study populations, because both blood type prevalence and ovarian reserve status differ among women of different races [14, 15].

**Table 4:** Summarized five studies on the relationship between ABO blood type and ovarian reserve

Study*	Year	Design	Sample size	Evaluation of ovarian reserve	Conclusions
Nejat et al.	2011	cross-sectional	544	FSH	A antigen (type A or AB) appears to be protective,
PMID: 21708793				DOR: FSH >10 IU/L	while type O appears to be associated with DOR
Mouzon et al.	2012	retrospective	1016	AMH	No association between blood type and ovarian reserve
PMID: 22411904					
Timberlake et al.	2013	cross-sectional	305	FSH	No association between blood type and DOR
PMID: 24055049				DOR: FSH >10 IU/L	
Sengul et al.	2014	prospective	500	FSH	No association between blood type and ovarian reserve
PMID: 25083178				DOR: FSH ≥10 IU/L	
Lin et al.	2014	retrospective	35479	FSH	Type O was less likely to have DOR, whereas B antigen
PMID: 25313097				DOR: FSH >10 IU/L	(type B or AB) was more likely to have DOR.

* Referenced studies are indexed for Medline are indicated (PMID).

Abbreviations: FSH, follicle stimulating hormone; DOR, diminished ovarian reserve; AMH, anti-mullerian hormone.

Our current findings demonstrated an association between blood type and ovarian reserve in Chinese women with subfertility, although they are also inconsistent with the above-mentioned studies. We found that blood type O was associated with a higher incidence and increased risk of DOR, which partially agrees with the conclusions of Nejat *et al*. Furthermore, we found that the A antigen (blood type A or AB) was not associated with ovarian reserve. Although both our study and Lin and colleagues examined Chinese subjects, the findings differ dramatically. This may be due to differences in blood type prevalence between the studies. The percentages of patients with blood type O (36.91%) were higher, while B (24.54 %) and AB (7.57%) were lower, in our study compared to Lin *et al*. Higher proportions of primary subfertility and ovarian surgery in our study might also contribute to this difference. Additional multi-center studies are needed to address these conflicting results.

Although the mechanism underlying the relationship between blood type and ovarian reserve is unknown, there are several possible explanations. The ABO gene locus is located on chromosome 9q34 and has three main allelic forms: the A allele, B allele, and O allele. The gene products of the ABO system are glycotransferases that catalyze the transfer of carbohydrates to the H antigen, which is a precursor of the ABO blood group antigens [16, 17]. The FSH and LH receptors are glycosylated proteins that are crucial for follicle development and maturation. The circulatory half-life and biologic activity of LH at the hormone receptor level are strongly affected by glycosylation [18]. Thus, it is likely that the biological activities of FSH and LH are altered by glycotransferases encoded by the O allele (those with blood type O lack the transferase enzyme), and that DOR is a consequence of this alteration. Genetic inheritance may also help explain the relationship between blood type and ovarian reserve. The nuclear receptor 5A1 (NR5A1) and transforming growth factor β receptor (TGFBR1) genes, which are located on chromosome 9q34 and 9q22 near the ABO locus, are related to ovarian function. Therefore, recombination may occur between these genes and the ABO type genes at a relatively high rate, which would increase the likelihood of these genes being inherited together with ABO [19, 20]. Additionally, variation in genes as phased haplotypes might affect the folding and levels of other proteins and thus increase the chances of inheriting specific allele combinations together [21, 22]. Furthermore, other genetic factors, such as FSH receptor polymorphisms [23] and fragile X mental retardation 1 gene (FMR1) permutation carrier status [24], are associated with elevated FSH levels. Additional studies are needed to explore whether these mechanisms contribute to associations between blood type and ovarian reserve.

The large sample size used in this study allowed us to assess the relationship between ABO blood type and ovarian reserve while adjusting for confounding factors associated with ovarian reserve, including age, AFC, BMI, subfertility type, and histories of endometriosis and ovarian surgery. Nevertheless, several limitations should be taken into consideration. First, FSH level alone was used to define DOR, although E2 was measured concomitantly to confirm that early follicular phase hormone levels were normal. Second, our study included only women seeking treatment for subfertility, limiting the applicability of our findings to the general population. Third, patient smoking history was not considered in this study, although smoking rates are low among Chinese women. Finally, AMH levels were not evaluated in our study, and future prospective studies are needed to clarify the association between blood type and AMH concentration.

In conclusion, we found that blood type O was a risk factor for DOR, while the B antigen (blood type B or AB) was a protective factor for ovarian reserve, in Chinese women with subfertility, suggesting that ABO blood type could be useful in evaluating ovarian reserve in clinical practice. Additional studies are needed to confirm this association and to identify the underlying mechanisms.

## MATERIALS AND METHODS

### Subjects

Women who underwent cycles of IVF or ICSI between February 2005 and May 2015 at the Reproductive Medicine Center of the First Affiliated Hospital of Wenzhou Medical University were included in this study. Participant data, including age, body mass index (BMI), blood type, duration and types of subfertility, history of previous pregnancies, history of endometriosis, history of ovarian surgery, and antral follicle count (AFC), was collected. Patients with missing data or who were more than 45 years old were excluded. A total of 14875 women were included in the analysis. The study was approved by the hospital ethics committee. Day-3 serum FSH was quantified using an auto immunoassay analyzer [Unicel Dxl 800, Beckman Coulter, USA]. The intra-assay coefficient of variation (CV) for FSH was 3.1%-4.3%, and the inter-assay CV was 4.3%-5.6%. DOR was defined as a day-3 serum FSH concentration > 10IU/L.

### Statistical analysis

Statistical analyses were performed using SAS version 8.1 (SAS Institute, Cary, NC). Continuous variables are shown as mean ± standard deviation (SD) and categorical variables are presented as counts with proportions. The study population was divided into two groups based on FSH level. Categorical data and continuous data were compared using the χ^2^ test and a non-parametric test, respectively, between the two groups. The association between blood type and DOR was evaluated using multiple logistic regression after adjusting for age, BMI, AFC, subfertility types, duration of subfertility, history of endometriosis and ovarian surgery, and blood type A.
